# Acute total hip arthroplasty with a highly-porous multi-holes cup in elderly patients after traumatic acetabular fracture: A case series and literature review

**DOI:** 10.1016/j.tcr.2024.101070

**Published:** 2024-06-09

**Authors:** Danilo Chiapale, Federico Vitali, Francesco Rubino, Marta Colombo, Matteo Formica

**Affiliations:** a Struttura Complessa di Ortopedia e Traumatologia, Ospedale S. Paolo, Savona, Italy; b Clinica Ortopedica, Ospedale Policlinico San Martino, Genova, Italy; c Clinical Department, Permedica SpA, Merate, Italy

**Keywords:** Acetabular fractures, Trauma, Acute THA, Highly-porous titanium

## Abstract

There are no general guidelines for the treatment of acetabular fractures. Open reduction and internal fixation is advised in young and active patients, while acute total hip arthroplasty (THA) is recommended for elderly patients in order to allow immediate weight bearing. Various THA systems have been reported.

We present four cases, mean age 79 years (range 67–92), of closed acetabular fractures managed with acute cementless THA, comprising a highly-porous multi-hole acetabular cup and a CLS-type femoral stem. After extensive pre-operative planning, autograft was used to fill in the acetabulum defects left by the trauma and the press-fit acetabular cup were implanted. One or more screws were used to improve primary stability and secure bone fragments. Patients were follow-up for mean 1.5 years (range 1.1–2.0). A Brooker III heterotopic ossification was the only complication occurred postoperatively. All the patients were satisfied, with a mean Harris Hip Score of 90 and Postel Merle D'Aubigné score of 16.3. There were no radiolucency lines nor osteolysis, showing that the cups were well osteointegrated and fractures united.

As the bone bed after acetabular fracture might be highly compromised, whenever acute THA is indicated, a highly-porous multi-hole cup could be used to limit radiolucency lines and aseptic loosening. The series is limited by the small number of cases but is significant for the promising results.

## Introduction

Acetabular fractures usually result from high-energy trauma. With the population living longer and more actively, osteoporotic acetabular fractures resulting from low-energy mechanisms are being seen more frequently. The incidence of these fractures per 100,000 patients increased from 3.67 in 2006 to 4.95 in 2016 in general population and from 17.06 to 23.18 in patients over 75 years [1]. The most appropriate treatment for acetabular fractures is still under debate. Open reduction and internal fixation (ORIF) has been shown to have better clinical results, while THA allows for immediate weight bearing and has lower reoperation rate [2]. To maximize the outcome of acute THA, the patients should be carefully selected. Elderly patients with acetabular fractures show broad heterogeneity and treatment should be highly individualised.

We reported four closed acetabular fractures managed with acute THA in elderly patients. Informed consent was obtained from all the patients or their next of kin. The relevant literature review about the last five years is also presented.

## Cases description

**Fig. 1:**
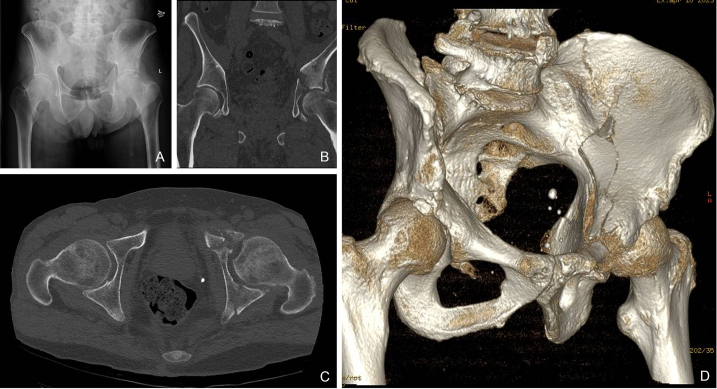
Preoperative plan images. (A) Antero-posterior X-ray, (B) (C) CT scans and (D) 3D reconstruction of the hip of case 4, who fractured its left acetabulum on both columns and injured the femoral head, after an accidental fall.

Between 2022 and 2023 at the Orthopaedic Trauma Department of the S. Paolo Hospital in Savona (Italy), 1 female and 3 males, mean age of 79 years (Table 1), underwent acute THA because of acetabular fracture. Patients were clinically evaluated in emergency room following a traumatic fall (Table 1). They reported pain, functional impairment, with the affected lower limb shortened and extra rotated. Preoperative planning was carried out using plain X-rays and CT scans (Fig. 1). Pre-existing coxarthrosis was diagnosed with grade between 3 and 4 on Kellgren-Lawrence system. Acetabular fractures were classified according to Judet-Letournel [3] and hips were evaluated for osteoporosis using the Singh Index [4]. Two out of four patients presented the gull-sign, indicative of dome impaction and harbinger of ORIF failure (Table 1). These are among the indications of poor prognosis in ORIF [5], therefore acute THA was scheduled. Patients were brought to the operation room averagely 1 day after trauma.

**Table 1:** Demographics of the patients and details of their fractures. It is also listed the number of screws used to increase cup fixation and secure bone fragments.

Gender	Age (years)	Kellgren Lawrence classification	Singh Index	Mechanism of injury	Judet Letorunel classification	Gull sign	Femoral head injury	Screws
1. Female	83	Grade IV	IV	Accidental fall	Anterior wall	Yes	Yes	2
2. Male	67	Grade IV	III	Bicycle fall	Anterior wall	No	Yes	1
3. Male	92	Grade III	IV	Accidental fall	Anterior wall and column	Yes	Yes	1
4. Male	73	Grade IV	III	Accidental fall	Both columns	No	Yes	4

**Fig. 2:**
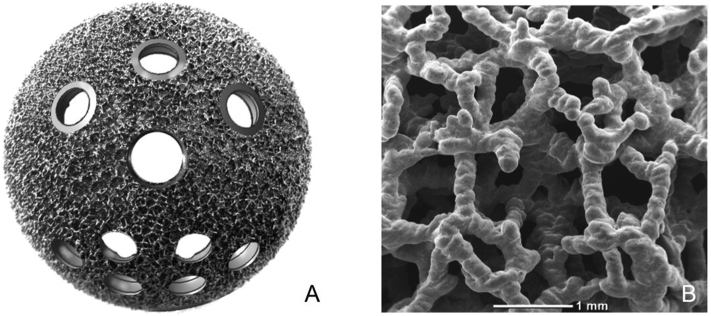
(A) JUMP SYSTEM TRASER® REVISION (Permedica, Merate, Italy) acetabular cup with (B) cancellous bone-like irregular titanium lattice on the bone side, additively manufactured in a one-step process together with the cup.

**Fig. 3:**
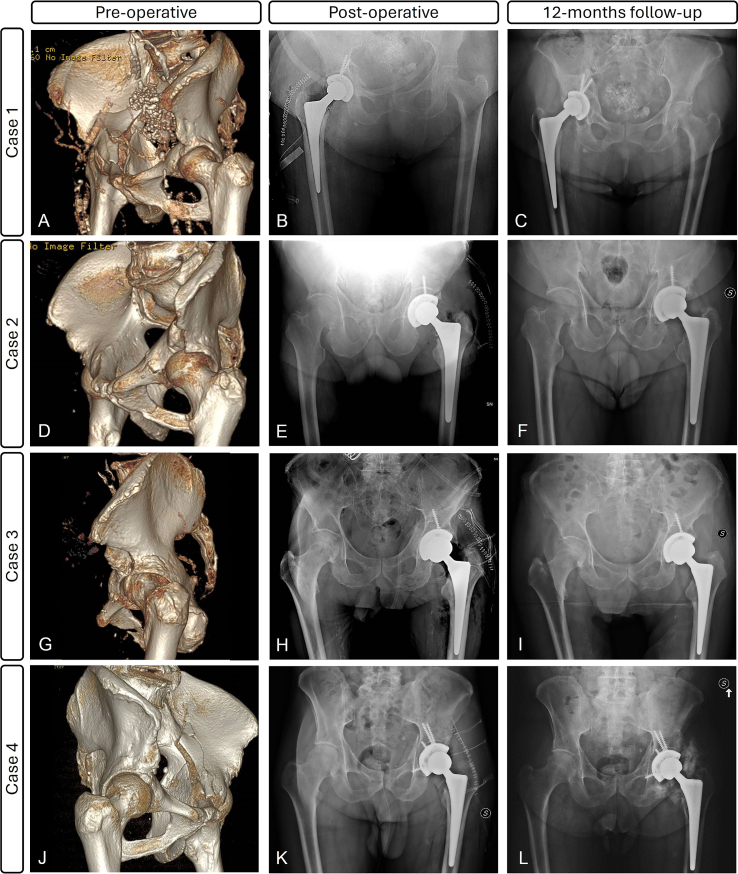
Preoperative 3D reconstruction images, postoperative and follow-up X-rays of the four cases. Case 1 received 2 screws into the ilium to fix the anterior wall fracture. For case 2 and case 3, one screw was deemed sufficient to increase the cup fixation and secure bone fragments. Case 4 had both columns fractured fixed with four screws. Brooker III heterotopic ossification was clearly visible at final follow-up.

Surgery was carried out under spinal anaesthesia, with the patient in lateral decubitus and following an extended postero-lateral approach. The fascia and extra-rotator muscles were resected to expose the hip and perform femoral neck osteotomy. Acetabular cavity was reamed as necessary. Autograft from the resected femoral head was put in place to fill in the defects caused by the injury. A cementless acetabular cup was fixed. Patients received a press-fit multi-hole acetabular cup (JUMP SYSTEM TRASER® REVISION, Permedica, Merate, Italy), featuring 3D-printed highly porous titanium on the bone side (Fig. 2). One or more screws were tightened in the ilium to increase stability. In particular, case 4, who fractured both columns, received three screws in the ilium and one in the ischium to secure bone fragments (Fig. 3). Then, femoral canal was broached, and a femoral stem implanted. The femoral component was a straight cementless stem CLS-type from the same manufacturer. Polyethylene acetabular insert and ceramic femoral head were placed. The hip was reduced, and stability verified. Extra-rotator muscles were re-inserted before layered suturing. THA position was checked with X-rays. Surgeries took about 55–65 min with a difference in haemoglobin levels ranging from −0.07 g/dL to 1.2 g/dL, between admission and discharge. No intraoperative complications were reported. As per protocol, low-molecular weight heparin was given to all patients and continued till the patient was mobilized. Patients were allowed to progressively weight-bear from day one and reached full weight-bearing on average on day seven. A patient got urosepsis and another one sustained anaemia, both resolved before leaving the hospital. Hospitalization lasted 10–17 days. Patients were discharged with indications of physiotherapy and postural exercises. Follow-ups were scheduled at 1, 3, 6 and 12 months. Patients were clinically evaluated for active and passive Range of Motion (ROM), ability to walk and satisfaction using Visual Analogue Scale (VAS), Harris Hip Score (HHS) and Postel Merle D'Aubigné (PMA) score. For each follow-up, antero-posterior and lateral X-rays were taken to assess cup position, its osteointegration, fracture recovery and any complication occurred.

Patients were followed-up for mean 1.5 years (range 1.1–2.0). At the latest follow-up available, patients were able to walk without any aid and went back to their daily life activities, with an overall good satisfaction and ROM gradually restored to pre-trauma condition. Only Case 4 had a functional limitation and was not able to tie his shoes. Nevertheless, all patients had a discrete muscle tonotrophism with no nerve injury. No residual leg length discrepancy was reported. Mean VAS was 3, mean HHS 90 (range 87–92) and mean PMA score 16.3 (range 16–17), showing “good” to “very good” ratings. Radiographically (Fig. 3), the four acetabular cups appeared well osteointegrated after the first month, with no radiolucency lines. The bone grafts were found stable, and the fractures were completely healed. A Brooker III heterotopic ossification (HO) was observed in Case 4, causing mild pain and limitation in daily life. No other complications nor failures were reported, but Case 3 died within a year from surgery.

## Discussion

We reported the results of four cases of elderly patients with an acetabular fracture that was managed with acute THA using a highly-porous multi-holes cup. Acetabular fractures are rare injuries in heterogeneous patient groups, making it difficult to develop adequately powered prospective studies. On one side, there is general consensus on managing these fractures with ORIF in young and active patients. On the other one, no guidelines have been definitively established for the management of acetabular fracture in the elderly [2]. Indications for acute THA, i.e. prognostic signs for ORIF failure, in the management of acetabular fractures include dome impaction (or “gull sign”), articular comminution, femoral head injury, pre-existing osteoarthritis or vascular necrosis, displaced femoral neck fracture, evidence of osteoporosis/osteopenia, and both column fracture [5]. The patients reported here received an acute THA due to their age and because of osteopenia, dome impaction, femoral head injury and type of fracture. In a recent meta-analysis, authors determined that 20 % of the patients reported a complication after acetabular fracture. The most common was HO, with a rate of 6.8 % for those clinically significant (Brooker grade III or IV). The rate of revision for acute THA was up to 4.3 % at 44 months follow-up [5]. Similar rate of revision, 4.9 % at 39 months follow-up, were reported in another article [6]. The main reason for revision identified were dislocation and aseptic loosening. Kelly et al. [7] noted the major complications that can occur after acute THA as dislocation (15.2 %), implant loosening (14.2 %) and infection (10.9 %). In a previous systematic review, De Bellis et al. [8] reported that radiographic loosening could reach a prevalence of 21 %, followed by dislocation (14 %). Another recurrent complication after acute THA for acetabular fracture is HO ranging from 10 % to 40% [8].

In more recent retrospective studies (Table 2), when considering acute THA performed with press-fit cups and screws or other fixation devices the main reported complications are those described above. Reason for revisions are infection, aseptic loosening and dislocation. Aprato et al. [9] reported the results of the 55 patients who received posterior plating and uncemented THA for displaced acetabular fractures. They found 15.8 PMA score (CI 14.9–16.8) comparable to our results. In this cohort, 3 cups were revised because of infection, dislocation, and aseptic loosening, respectively. Another infection and two aseptic loosening were recorded in other studies [10,11]. Cup radiographic loosening was 6.2% [12]–9.5% [13]. The reported cases were asymptomatic. HO was the most common complication, Smakaj et al. observed 3 HO (14.3 %) not requiring further surgery, in the acute fix and replace group.

**Table 2:** Overview of the relevant literature, from the last 5 years about uncemented acute THA. (FU: follow-up; OHS: Oxford Hip Score; HHS: Harris Hip Score; PMA: Postel Merle d'Aubigné; HO: heterotopic ossification; DVT: deep vein thrombosis; PE: pulmonary embolism; LLD: leg length discrepancy).

Reference	Acute THA	Age (y)	Additional acetabular fixation	FU months	Clinical score	Complications	Revisions
Malhotra 2019 [16]	18	46.4	Screws	57.6	91 HHS	Infection 1, sciatic nerve palsy 1, dislocation 1, LLD 2, HO 2	None
MacCormick 2019 [10]	16	56.4	Plates and screws	81.6	44 OHS	Pneumonia 1, urosepsis 1	Aseptic loosening 1, infection 1
Aprato 2020 [9]	55	71 (65–84)	Plates and screws	27	15.8 PMA	Sciatic nerve impairment 1, DVT 1, PE 1	Infection 1, dislocation 1, aseptic loosening 1
Becker 2020 [21]	10	74	Cage and screws	12	72.0 HHS	None	None
Sarantis 2020 [12]	16	80.1 (76–89)	Cage and screws	71.8	88.3 HHS	DVT 1, wound infection 1, dislocation 1, cup radiographic loosening 1, HO 2	None
Nicol 2021 [11]	12	87 (68–93)	Cage and screws	60	40.1 OHS	HO 2	Infection 1
Chen 2021 [22]	8	77 (59–89)	Plates and screws	12	15.8 PMA	HO 1	None
Selvaratnam 2021 [23]	7	67 (48–94)	Plates and screws	43	–	PE 1	None
Smakaj 2022 [13]	21	73.4 (±1.84)	Plates and screws	24	83.8 HHS	Cup radiographic loosening 2, HO 3, wound infection 3, DVT, 3, others 1	None

Solomon et al. [14] prospectively analysed cup migration of eleven acute THA with cup-cage reconstruction after acetabular fracture in elderly. They inserted tantalum beads in stable parts of the ilium and ischium to perform radiostechiometric analysis (RSA). At 1 year, three acetabular components had proximal migration or sagittal rotation above the suggested limits of migration for primary THA predicting loosening. Cup migration was stable at 2 years. None of these patients were symptomatic nor required revision. There are no RSA studies about acetabular fractures managed with highly-porous cups without any additional cage or plate.

Even if in patients aged more than 65 years, management of acetabular fractures with THA increased 16.5% [15], achieving acetabular component stability remains challenging. Only one article documented the use of a highly porous multi-hole cup in acetabular fractures [16] in young patients, unlike other retrieved studies. Similarly to our series, the reported mean HHS was 90 (range 80–96) and acetabular cups were well integrated with no radiolucency lines. They noted two cases of HO, one asymptomatic and another one causing limp. We observe one HO causing functional limitations.

Even though acute THA seems straightforward, it has the intrinsic challenge of obtaining long-term acetabular component fixation. The bone bed may not be adequate and additional fixation may be required to provide primary stability. Modern highly porous metal cups have demonstrated rapid and enhanced biologic fixation of the acetabular cups into the bone [17]. The 3D-printed cancellous bone-like irregular titanium lattice demonstrated early osteointegration properties and excellent stability [18]. Moreover, the presence of six additional holes allows for supplementary screws fixation. Even if there is paucity on the use of revision porous cups to treat acetabular fractures, this study is supported by the excellent results of porous cups in primary elective THA [19,20]. Making this cup suitable and effective for acetabular fracture treatment with acute THA.

## Conclusion

This retrospective case series is limited by the limited number of cases. Nevertheless, our promising clinical and radiographic results support the use of highly porous multi-hole acetabular cups in the acute management of acetabular fractures in a selected cohort of elderly patients. Further investigation could involve more patients and the use of an accurate method to measure cup migration, such as RSA.

## Funding

This research did not receive any specific grant from funding agencies in the public, commercial, or not-for-profit sectors.

## CRediT authorship contribution statement

**Danilo Chiapale:** Writing – review & editing, Writing – original draft, Validation, Supervision, Resources, Project administration, Methodology, Formal analysis, Data curation, Conceptualization. **Federico Vitali:** Writing – review & editing, Writing – original draft, Validation, Supervision, Project administration, Methodology, Formal analysis, Data curation, Conceptualization. **Francesco Rubino:** Writing – review & editing, Writing – original draft, Validation, Supervision, Resources, Project administration, Methodology, Formal analysis, Data curation, Conceptualization. **Marta Colombo:** Writing – review & editing, Writing – original draft, Supervision, Project administration, Methodology, Conceptualization. **Matteo Formica:** Writing – review & editing, Validation, Supervision, Methodology.

## Declaration of competing interest

MC is paid employee as clinical researcher for Permedica. Other authors declare no conflict of competing interest.

## References

[1] Melhem E., Riouallon G., Habboubi K., Gabbas M., Jouffroy P. (2020). Epidemiology of pelvic and acetabular fractures in France. Orthop. Traumatol. Surg. Res..

[2] Giustra F., Cacciola G., Pirato F. (2024). Indications, complications, and clinical outcomes of fixation and acute total hip arthroplasty for the treatment of acetabular fractures: a systematic review. Eur. J. Orthop. Surg. Traumatol..

[3] Letournel E., Judet R., Elson R.A., Letournel E., Judet R., Elson R.A. (1993). Fractures of the Acetabulum.

[4] Singh M., Nagrath A.R., Maini P.S. (1970). Changes in trabecular pattern of the upper end of the femur as an index of osteoporosis. J. Bone Joint Surg. Am..

[5] Jauregui J.J., Weir T.B., Chen J.F. (2020). Acute total hip arthroplasty for older patients with acetabular fractures: a meta-analysis. J. Clin. Orthop. Trauma.

[6] Capone A., Peri M., Mastio M. (2017). Surgical treatment of acetabular fractures in the elderly: a systematic review of the results. EFORT Open Rev..

[7] Kelly M., Peterson D.F., Yoo J., Working Z.M., Friess D., Kagan R. (2023). Risk of revision and complications after total hip arthroplasty for acute treatment of acetabular fracture. J. Arthroplast..

[8] De Bellis U.G., Legnani C., Calori G.M. (2014). Acute total hip replacement for acetabular fractures: a systematic review of the literature. Injury.

[9] Aprato A., Giachino M., Messina D., Massé A. (2020). Fixation plus acute arthroplasty for acetabular fracture in eldery patients. J. Orthop..

[10] MacCormick L.M., Lin C.A., Westberg J.R., Schmidt A.H., Templeman D.C. (2019). Acute total hip arthroplasty versus open reduction internal fixation for posterior wall acetabular fractures in middle-aged patients. OTA Int..

[11] Nicol G.M., Sanders E.B., Kim P.R., Beaulé P.E., Gofton W.T., Grammatopoulos G. (2021). Outcomes of total hip arthroplasty after acetabular open reduction and internal fixation in the elderly-acute vs delayed total hip arthroplasty. J. Arthroplast..

[12] Sarantis M., Stasi S., Milaras C., Tzefronis D., Lepetsos P., Macheras G. (2020). Acute total hip arthroplasty for the treatment of acetabular fractures: a retrospective study with a six-year follow-up. Cureus.

[13] Smakaj A., Rovere G., Scoscina D. (2022). Outcomes of acetabular fractures treated with acute fix and replace versus open reduction and internal fixation in elderly population: a multicentric retrospective study. Int. Orthop..

[14] Solomon L.B., Studer P., Abrahams J.M. (2015). Does cup-cage reconstruction with oversized cups provide initial stability in THA for osteoporotic acetabular fractures?. Clin. Orthop. Relat. Res..

[15] Patterson J.T., Wier J., Kumaran P., Adamczyk A. (2023). Rising incidence of acute total hip arthroplasty for primary and adjunctive treatment of acetabular fracture in older and middle-aged adults. Eur. J. Orthop. Surg. Traumatol..

[16] Malhotra R., Gautam D. (2019). Acute total hip arthroplasty in acetabular fractures using modern porous metal cup. J. Orthop. Surg. (Hong Kong).

[17] Malahias M.A., Kostretzis L., Greenberg A., Nikolaou V.S., Atrey A., Sculco P.K. (2020). Highly porous titanium acetabular components in primary and revision total hip arthroplasty: a systematic review. J. Arthroplast..

[18] Ragone V., Canciani E., Arosio M. (2020). In vivo osseointegration of a randomized trabecular titanium structure obtained by an additive manufacturing technique. J. Mater. Sci. Mater. Med..

[19] Familiari F., Barone A., De Gori M. (2024). Short- to mid-term clinical and radiological results of selective laser melting highly porous titanium cup in primary total hip arthroplasty. J. Clin. Med..

[20] Ciriello V., La China R., Chirillo D.F. (2023). Is modular dual mobility superior to standard bearings for reducing dislocation risk after primary total hip arthroplasty? A retrospective comparative multicenter study. J. Clin. Med..

[21] Becker C.A., Linhart C., Bruder J. (2021). Cementless hip revision cup for the primary fixation of osteoporotic acetabular fractures in geriatric patients. Orthop. Traumatol. Surg. Res..

[22] Chen M.J., Wadhwa H., Bellino M.J. (2021). Sequential ilioinguinal or anterior intrapelvic approach with anterior approach to the hip during combined internal fixation and total hip arthroplasty for acetabular fractures. Eur. J. Orthop. Surg. Traumatol..

[23] Selvaratnam V., Panchani S., Jones H.W., Chitre A., Clayson A., Shah N. (2021). Outcomes of acute fix and replace in complex hip posterior fracture dislocations with acetabular fractures: a minimum of 3 years follow-up. Acta Orthop. Belg..

